# The Effects of Tissue Flossing on Ankle Dorsiflexion Range of Motion, Balance, and Gait in Participants with Limited Ankle Dorsiflexion: A Randomized Controlled Trial

**DOI:** 10.3390/medicina62050906

**Published:** 2026-05-07

**Authors:** Byoung-Hyoun Moon, Ji-Won Kim

**Affiliations:** 1 Department of Physical Therapy, Cheongam University, 1641 Noksaek-ro, Suncheon-si 57997, Jeollanam-do, Republic of Korea; mbh930@naver.com; 2 Department of Physical Therapy, Nambu University, 23, Cheomdanjungang-ro, Gwangsan-gu, Gwangju 62271, Republic of Korea

**Keywords:** ankle dorsiflexion, balance, gait, tissue flossing

## Abstract

*Background*: Tissue flossing has been recommended to enhance ankle dorsiflexion (DF), jump performance, and agility; however, limited evidence exists regarding its effects on balance and gait. *Objective*: This study aimed to investigate the effects of tissue flossing on ankle DF, balance, and gait in participants with limited ankle DF. *Methods*: Forty participants with restricted DF were assessed at baseline and then randomly assigned to either an intervention group (*n* = 20) or a control group (*n* = 20) using a computerized randomization tool. The intervention group underwent tissue flossing using a floss band (Sanctband COMPRE Floss™ Lime), while the control group performed low-intensity active DF and plantarflexion exercises for 2 min. Ankle DF, sway area during the one-leg test (OLT), and gait parameters, including foot strike (FS) and toe-off angles, were measured before and after the intervention. *Results*: The intervention group showed significantly greater improvements in DF, OLT, and FS compared to the control group. *Conclusions*: Tissue flossing is an effective intervention for improving ankle DF, balance, and gait in individuals with restricted DF. Clinical trial registration: ClinicalTrials.gov (identifier: NCT06708052; registered on 25 November 2024; registered retrospectively).

## 1. Introduction

Sufficient ankle joint range of motion (ROM) is a fundamental biomechanical requirement for the execution of efficient and safe locomotor tasks [1]. Approximately 10° of dorsiflexion (DF) is required during the terminal stance phase of a normal gait, and 15–20° of plantarflexion (PF) is necessary during heel-off to ensure proper forward propulsion and limb clearance [2]. Activities that involve higher neuromechanical demands, such as running or cutting, require even greater DF due to increased anterior displacement of the tibia over the talus [3,4].

When ankle mobility is restricted, the resulting alterations in lower-limb kinematics may lead to suboptimal loading patterns, joint instability, and impaired force transmission across the kinetic chain [5,6]. These biomechanical changes increase the risk of musculoskeletal pathologies, including lateral ankle sprains, gastrocnemius strains, and Achilles tendinopathy [7]. Moreover, limited ankle DF may provoke compensatory movement strategies in proximal joints, such as the knee or lumbar spine, further predisposing individuals to injury [8,9,10]. Impaired ankle mobility also diminishes proprioceptive acuity and neuromuscular control, which are key components of postural stability and fall prevention [11].

Restoring adequate ankle DF ROM is therefore a critical goal in musculoskeletal rehabilitation and sports performance enhancement. Maintaining and improving ankle mobility requires sufficient fascial flexibility and soft tissue extensibility [12]. Common interventions include myofascial release, joint mobilization, and neurodynamic mobilization [7,13]. However, these methods are passive and clinician-dependent, often necessitating adjunctive active interventions to promote motor learning and ensure transfer to dynamic functional tasks [14].

In response to this clinical need, tissue flossing has emerged as a novel, active, compression-based technique. It involves tightly wrapping a thick elastic band (tissue floss) around a joint segment while the individual simultaneously performs active movement patterns [15]. The resulting compressive and shearing forces are theorized to alter tissue viscosity, promote myofascial gliding, enhance circulation, and stimulate mechanoreceptors, thereby facilitating improvements in ROM, proprioception, and neuromotor control [16].

Emerging evidence supports the acute functional benefits of tissue flossing. Driller and Overmayer [17] reported immediate improvements in ankle ROM, lunge capacity, jump height, and movement velocity following its application in conjunction with active motion. Similarly, Huang et al. [18] found that applying tissue flossing directly to the ankle produced greater increases in DF and agility than application to the proximal calf, with effects persisting for up to 1 h. Kaneda et al. [19] demonstrated that tissue flossing applied to the gastrocnemius muscle resulted in greater improvements in DF ROM and rate of force development compared to static stretching, suggesting superior efficacy of tissue flossing in enhancing both flexibility and muscle performance.

However, the current literature is largely limited to healthy populations and short-term performance outcomes. Evidence regarding the effects of tissue flossing on balance and gait remains limited, particularly in individuals with restricted ankle DF. Furthermore, the impact of tissue flossing on balance and gait parameters remains inadequately investigated, despite the relevance of these parameters to both injury prevention and performance optimization.

Therefore, this study aimed to examine the immediate effects of a single tissue flossing intervention on ankle DF ROM, balance, and gait parameters in individuals with limited DF. We hypothesized that tissue flossing would result in greater improvements in ankle DF ROM, balance, and gait parameters compared to active exercise alone. These findings may provide evidence to support clinical decision-making regarding the integration of active compression techniques into ankle mobility programs.

## 2. Materials and Methods

### 2.1. Participants

A total of 40 participants who met the eligibility criteria voluntarily enrolled in this study. The sample size estimation was exploratory due to the lack of prior studies reporting effect sizes for this specific intervention. Study inclusion required participants to demonstrate <10° of ankle DF during the baseline assessment, as measured using standard goniometry, which has been previously reported as a criterion indicating limited ankle dorsiflexion range of motion [9,20]. Exclusion criteria were a history of orthopedic injury to the lower extremities within the past year, impaired balance, previous lower-limb surgery, visual impairments, dermatological conditions, or any ongoing treatment targeting the calf muscles. All participants provided written informed consent prior to participation, in accordance with the Declaration of Helsinki. The study protocol was approved by the Institutional Review Board of Nambu University (IRB No. 1041478-2022HR-008). Participant recruitment began in July 2022, and the study was subsequently registered in the ClinicalTrials.gov database (identifier: NCT06708052; registered on 25 November 2024). The delay in registration was due to a lack of awareness of prospective registration requirements at the time of study initiation, and the trial was therefore registered retrospectively to ensure transparency.

### 2.2. Study Procedure and Measurements

All study procedures were thoroughly explained to participants before enrollment, and written informed consent was obtained from each individual. Baseline assessments were conducted to measure ankle DF ROM, balance, and gait parameters, specifically foot strike (FS) and toe-off (TO) angles. Following the baseline evaluation, participants were randomly assigned to either the intervention group (n = 20) or the control group (n = 20) using a computerized randomization tool (http://www.randomizer.org). This study was designed as a single-blinded randomized controlled trial. Participants were not informed of their group allocation or the specific differences between the intervention and control conditions. Both groups performed similar active ankle exercises, and the control condition was designed to resemble a plausible intervention to minimize participants’ ability to distinguish between groups. All outcome measurements were performed by a single experienced examiner using a standardized protocol.

**Figure 1:**
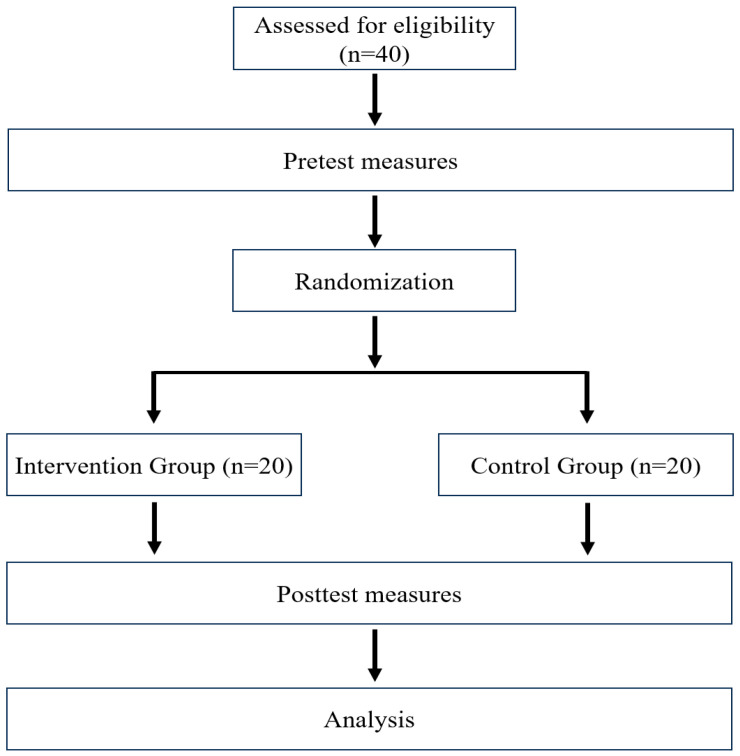
Flow chart of the study.

The intervention group performed active ankle exercises in combination with tissue flossing, whereas the control group engaged in the same exercises without the flossing component. Immediately after the intervention, all participants underwent post-intervention assessments using the same procedures as those employed during baseline testing, including measurements of ankle DF ROM, balance, and gait parameters (Figure 1).

#### 2.2.1. Ankle DF ROM

Ankle DF ROM was assessed using a standard universal goniometer. Participants were positioned in a supine posture with the knee fully extended. For passive ROM assessment, the examiner placed one hand on the plantar surface of the forefoot, maintained the subtalar joint in a neutral position, and applied pressure until a firm end-feel was perceived. The axis of the goniometer was aligned with the lateral malleolus; the stationary arm was aligned along the fibular shaft directed toward the fibular head, and the movable arm was positioned parallel to the fifth metatarsal [21]. Each measurement was performed thrice, and the mean value was used for statistical analysis. The intrarater reliability of the passive ankle DF had an intraclass correlation coefficient (ICC) of 0.92–0.96 [22].

#### 2.2.2. Balance Ability

Balance ability was evaluated using the one-leg test (OLT) in conjunction with a wireless inertial measurement unit (APDM Inc., Portland, OR, USA). To quantify postural sway, participants performed the test for 30 s. Three Opal sensors were secured using elastic straps to both ankles and to the lumbar spine at the fifth lumbar vertebra (L5). Kinematic data were sampled at a frequency of 128 Hz, processed in real time, and transmitted wirelessly to a laptop using Mobility Lab™ software version 2.0 (Mobility Lab™, Arlington, VA, USA).

The test began with participants standing upright with both feet flat on the ground and their gaze directed forward. Upon verbal instruction, participants shifted their weight onto the dominant leg, crossed both arms over the chest, and raised the non-dominant leg, flexing both the hip and knee joints to approximately 90°. Following an auditory cue, they attempted to maintain an OLT for the entire 30 s duration. Trials were considered invalid and repeated if the elevated foot contacted the ground before the trial was completed.

#### 2.2.3. Gait Parameters

Gait parameters were assessed using the same wireless inertial measurement system (APDM Inc., Portland, OR, USA) used for balance evaluation. This system, equipped with Opal sensors, sampled kinematic data at 128 Hz and wirelessly transmitted the data to a laptop for real-time analysis using Mobility Lab™ software (Mobility Lab™, Arlington, VA, USA). Three sensors were securely fastened with elastic straps: one on each ankle and one over L5.

Participants performed the 2 min walk test barefoot along a 10 m straight path in a quiet indoor corridor. At the start of the test, participants stood motionless at the starting line and began walking at a self-selected, comfortable pace upon hearing an auditory cue. The task concluded with a second auditory cue after 2 min. Gait metrics, including FS and TO angles, were extracted from the recorded data for subsequent analysis.

### 2.3. Intervention

Tissue flossing was administered according to a standardized protocol by a certified clinician. A lime green elastic band (Sanctband COMPRE Floss™, 2 in × 3.5 m; Shah Alam, Malaysia) composed of natural rubber was used for the procedure. The band was stretched to approximately 1.5 times its resting length and applied to the affected ankle with 50% overlap.

**Figure 2:**
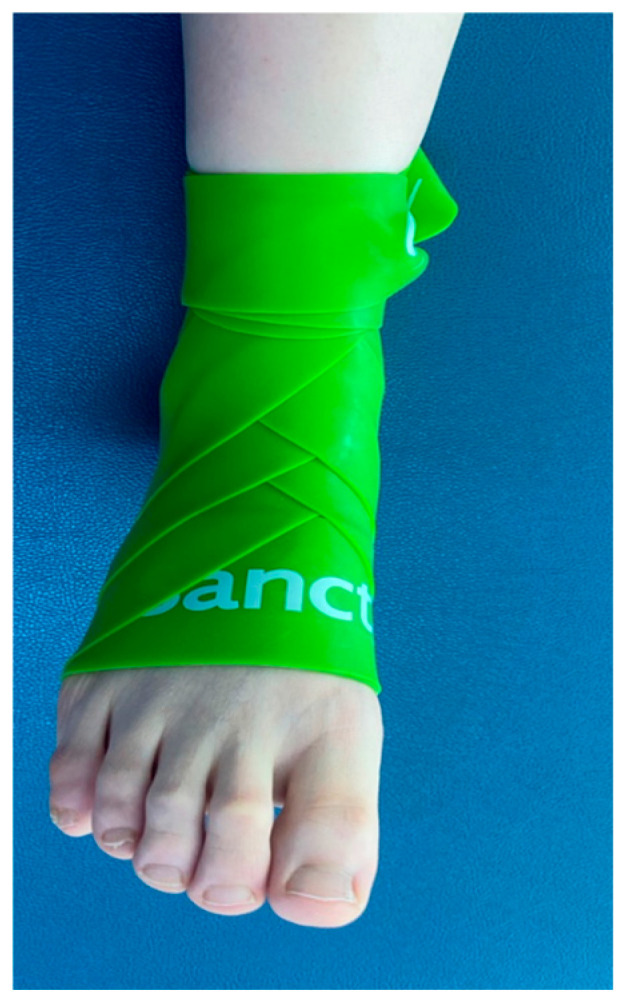
Tissue flossing intervention.

Wrapping began at the level of the fifth metatarsal, proceeded with two horizontal turns across the dorsum of the foot, and continued toward the medial malleolus. The band was then passed posterior to the Achilles tendon and around the lateral malleolus, forming a figure-eight pattern. Wrapping was completed after three full loops, and the band was secured with two additional turns around the lateral malleolus (Figure 2).

Following the wrapping procedure, participants performed low-intensity active DF and PF exercises for 2 min. The band was then removed, and participants were instructed to walk lightly on flat ground for approximately 1 min to facilitate reperfusion and restore normal circulation.

The control group performed the same low-intensity active DF and PF exercises for 2 min without the application of tissue flossing. Thereafter, participants were also instructed to walk at a light pace on level ground for approximately 1 min.

### 2.4. Statistical Analyses

Statistical analyses were conducted using SPSS version 22.0 for Windows (IBM Corp., Armonk, NY, USA). Descriptive statistics were calculated for participant demographics, including age, height, weight, and body mass index, and are presented as means ± standard deviation. The normality of all outcome variables was assessed using the Kolmogorov–Smirnov test.

Two-way repeated-measures analysis of variance was performed to evaluate the main effects of group (intervention vs. control) and time (pre- vs. post-intervention), and the interaction effects. When significant main or interaction effects were identified, post hoc comparisons were conducted using paired or independent *t*-tests, as appropriate. Statistical significance was set at *p* < 0.05.

## 3. Results

A total of 40 participants with limited ankle DF ROM were included in this study. The mean age was 29.7 ± 3.6 years and 29.8 ± 4.0 years in the intervention and control groups, respectively. The average height was 169.1 ± 6.8 cm in the intervention group and 170.2 ± 7.3 cm in the control group, while the average weight was 70.0 ± 17.0 kg and 69.8 ± 15.3 kg, respectively. No statistically significant differences were observed between the two groups in demographic characteristics (*p* > 0.05). There were no dropouts, and all 40 participants completed the intervention and were included in the final analysis (Table 1).

A significant time-by-group interaction effect was observed for ankle DF ROM (F = 129.408, *p* < 0.001; Table 2). Ankle DF ROM increased significantly from pre- to post-intervention in both the intervention group (*p* < 0.001) and the control group (*p* = 0.004). However, the intervention group demonstrated a significantly greater improvement (*p* < 0.001).

For OLT, a significant time-by-group interaction was also identified (F = 9.193, *p* = 0.007; Table 2). Both the intervention group (*p* < 0.001) and the control group (*p* = 0.014) showed significant improvements from pre- to post-intervention. However, the between-group difference in OLT performance was not statistically significant (*p* = 0.12).

A significant time-by-group interaction was also found for the FS angle (F = 33.285, *p* < 0.001; Table 2). The FS angle increased significantly from pre- to post-intervention in the intervention group (*p* < 0.001), and the intervention group demonstrated significantly greater gains in FS compared to the control group (*p* = 0.015).

No significant time-by-group interaction was observed for the TO angle (F = 1.815, *p* = 0.194; Table 2). Within-group analysis revealed a significant improvement in TO angle in the intervention group (*p* = 0.020), whereas no significant change was observed in the control group (*p* = 0.617). However, the between-group difference in TO change was not statistically significant (*p* = 0.194).

## 4. Discussion

In this study, tissue flossing was applied to the ankle joints of individuals with limited DF ROM. Following the intervention, the tissue flossing group demonstrated significantly greater improvements in DF ROM, OLT performance, and FS during gait compared to the control group. These findings suggest that the tissue flossing intervention may be associated with improvements in ankle mobility, balance, and gait parameters in individuals with restricted DF. Although the TO angle increased in both groups from pre- to post-intervention, the change was not statistically significant. However, the magnitude and clinical relevance of these changes should be interpreted with caution.

Adequate ankle DF during gait requires adequate extensibility and mobility of periarticular soft tissues [23]. In the present study, DF ROM increased significantly in both the intervention and control groups; however, the magnitude of improvement was greater in the intervention group. This enhanced response may be attributed to the application of the tissue flossing technique, which integrates external compression with active or passive movement, potentially facilitating the reduction in adhesions between the skin, fascia, and underlying musculature [24,25], improving sensory–motor integration. These outcomes are consistent with previous research. Stevenson et al. [26] reported immediate improvements in ankle ROM and performance on the weight-bearing lunge test following the application of tissue flossing with active movement. Similarly, Sano et al. [27] found that a brief session of calf tissue flossing combined with voluntary isometric contractions resulted in significant and immediate increases in ankle DF ROM and PF torque in healthy adults, suggesting that tissue flossing can effectively enhance joint mobility and muscle function even after a short application. Thus, tissue flossing appears to be a potential intervention for improving ankle function in individuals with limited DF. However, although a significant between-group difference was observed, the magnitude of change was relatively small and may fall below the minimal detectable change, suggesting limited clinical relevance.

Regarding balance, the sway area during the OLT significantly decreased in both groups, with a more pronounced reduction observed in the intervention group compared to the control group. This finding may reflect improved postural stability and neuromuscular control. This finding may reflect improved postural stability and neuromuscular control. Ankle DF plays a critical role in maintaining proper biomechanical alignment of the center of mass over the base of support during the OLT, thereby contributing to static postural stability [28]. Improvements in DF ROM may reduce compensatory movement patterns, such as excessive foot pronation, hip external rotation, and lateral trunk sway, resulting in a more biomechanically efficient and stable posture [29]. In addition, the application of tissue flossing has been suggested to provide augmented sensory input through stimulation of cutaneous and articular mechanoreceptors [30,31], which may be associated with enhanced proprioceptive feedback and motor control. However, despite these statistically significant findings, the clinical significance of the observed changes in OLT performance remains uncertain and should be interpreted with caution.

In terms of gait parameters, the intervention group demonstrated a greater increase in the FS angle compared to the control group. This finding suggests that tissue flossing may be associated with improvements in initial contact mechanics during walking in individuals with limited ankle DF. Restrictions in ankle DF are known to impair normal heel-strike mechanics, often resulting in compensatory gait strategies such as forefoot or midfoot initial contact, excessive foot pronation, and early heel rise [32]. These abnormal gait patterns can disrupt the kinetic chain of the lower extremity, reduce movement efficiency, and increase the risk of musculoskeletal injuries [33]. Improvements in the FS angle may therefore reflect a shift toward a more typical gait pattern. However, the extent to which these changes translate into meaningful functional improvements or reduced injury risk remains unclear and warrants further investigation.

In contrast, no significant time-by-group interaction was observed for the TO angle. This may be explained by the multifactorial nature of TO mechanics, which depend not only on ankle joint mobility but also on plantarflexor strength and coordination of the lower-limb kinetic chain [34,35]. A single session of tissue flossing may not be sufficient to influence these factors to a measurable extent. These findings suggest that the effects of tissue flossing may be more pronounced during early stance phase mechanics rather than propulsion-related variables.

This study had several limitations. First, the intervention consisted of a single session, limiting the ability to assess long-term effects. Second, the sample consisted of healthy young adults, which restricts generalizability to clinical populations. Third, a sham or attention-matched control condition was not included, limiting the ability to account for placebo effects. Fourth, only short-term outcomes were assessed. Fifth, the study was registered retrospectively, which may raise concerns regarding transparency and potential bias in study design and reporting. Sixth, no formal a priori sample size calculation was performed; therefore, the sample size should be interpreted with caution, as it was determined on an exploratory basis. Finally, although efforts were made to blind participants, complete blinding may not have been fully achieved due to the perceptible nature of the intervention.

## 5. Conclusions

This study aimed to investigate the effects of tissue flossing on ankle DF, balance, and gait parameters in individuals with restricted ankle DF. The findings suggest that tissue flossing may be associated with short-term improvements in ankle DF ROM, OLT performance, and FS angle. However, given the acute nature of the intervention and the modest magnitude of the observed changes, these results should be interpreted with caution. Tissue flossing may be considered a potential adjunct strategy for improving ankle function. However, further research with larger sample sizes, repeated interventions, and long-term follow-up is required to determine its clinical relevance.

## Figures and Tables

**Table 1:** Physical characteristics of the participants.

Variable (Unit)	Intervention Group (n = 20)	Control Group (n = 20)	t	*p*
Age (yr)	29.7 ± 3.6	29.8 ± 4.0	0.083	0.934
Height (cm)	169.1 ± 6.8	170.2 ± 7.3	0.493	0.625
Weight (kg)	70.0 ± 17.0	69.8 ± 15.3	0.039	0.969
BMI (kg/m^2^)	24.2 ± 4.4	24.2 ± 5.3	0.033	0.974

Values are presented as mean ± standard deviation, BMI: body mass index.

**Table 2:** Pre- and post-intervention measurements in both groups.

Variable/Group	Pre-Test ^a^	Post-Test ^a^	Factor	F	*p*
DF (°)					
Intervention group	6.30 ± 2.43	10.65 ± 2.21	Time	226.190	0.000 *
			Group	8.478	0.009 *
Control group	7.20 ± 1.47	7.85 ± 1.35	T × G	129.408	0.000 *
OLT (mm^2^)					
Intervention group	269.71 ± 127.03	128.26 ± 63.19	Time	63.766	0.000 *
			Group	0.096	0.760
Control group	213.47 ± 110.90	164.17 ± 77.60	T × G	9.193	0.007 *
FS (°)					
Intervention group	16.85 ± 5.44	22.20 ± 5.31	Time	41.036	0.000 *
			Group	1.894	0.185
Control group	17.33 ± 5.54	17.60 ± 6.05	T × G	33.285	0.000 *
TO (°)					
Intervention group	23.35 ± 3.35	24.90 ± 3.46	Time	4.307	0.052
			Group	3.240	0.088
Control group	21.80 ± 5.10	22.14 ± 4.80	T × G	1.815	0.194

*: *p* < 0.05. Abbreviations: DF: dorsiflexion, OLT: one-leg test, FS: foot strike, and TO: toe-off. ^a^ Values are means ± SD. T × G: Time × Group.

## Data Availability

The data that support the findings of this study are available from the corresponding author upon request.

## Abbreviations

The following abbreviations are used in this manuscript:

DF	Dorsiflexion
ROM	Range of motion
OLT	One-leg test
FS	Foot strike
TO	Toe-off
